# The Psychometric Properties of Attentional Control Scale and Its Relationship with Symptoms of Anxiety and Depression: A Study on Iranian Population

**Published:** 2017-04

**Authors:** Imaneh Abasi, Parvaneh Mohammadkhani, Abbas Pourshahbaz, Behrouz Dolatshahi

**Affiliations:** Department of Clinical Psychology, University of Social Welfare and Rehabilitation Sciences, Tehran, Iran.

**Keywords:** *Anxiety*, *Attention*, *Emotion*, *Depression*, *Psychometrics*

## Abstract

**Objectives:** The attentional control scale is a self- report questionnaire that assesses individual differences in attentional control. Despite its extensive use, the psychometric properties of the Persian version of the ACS are not well understood. Thus, the present study aimed at investigating the psychometric properties of the attentional control scale and its relationship with symptoms of anxiety and depression in Iranian population.

**Method:** Using quota sampling, we asked a community sample of 524 to respond to Attentional Control Scale, mindfulness, emotion regulation, social anxiety, depression, generalized anxiety, worry, and rumination. SPSS (Version 23) was used for data analysis.

**Results:** Exploratory factor analysis yielded 2 factors of focusing and shifting, which accounted for 30.93% of the total variance. The results of convergent validity revealed that reappraisal, as an emotion regulation strategy and mindfulness facets, had a positive relationship with focusing, shifting, and the total score of the attentional control scale. Furthermore, worry, rumination, depression, generalized anxiety, and social anxiety symptoms all had negative relationships with focusing, rumination, and the total score of the attentional control scale. In addition, the results of incremental validity revealed that focusing, not shifting, uniquely predicted depression and generalized anxiety symptoms. Furthermore, both focusing and shifting uniquely predicted social anxiety symptoms. Test- retest reliability of focusing and shifting was 0.80 and 0. 76, respectively.

**Conclusion:** Attentional control scale has been demonstrated to have acceptable validity and reliability in Iranian population. However, further studies are needed to evaluate other aspects of the ACS like CFA.

Attention and attentional control are 2 mainly related constructs associated with emotion regulation, and are considered as emotion regulation strategies in anxiety and mood disorders (1-3). Attentional control as voluntary control of emotion is central to emotion regulation, as the process model of emotion regulation posits that individuals regulate their emotion in the situation-attention-appraisal-response sequence and that attentional deployment is a cardinal emotion regulation strategy in developing and maintaining emotional disorders (4, 5).

Attentional bias towards negative stimuli or information interferes with the flexible use of emotion regulation strategies and contributes to depression (6).

Over the last 3 decades, most studies have highlighted the role of attentional biases and attentional control in psychopathology including anxiety (7), social anxiety disorder (8), trait anxiety (9), depression (10), generalized anxiety disorder (11), rumination (12), and worry (13); and various treatments have been developed to control them in people suffering from several anxiety and depression symptoms (14-16).

Attentional control is regulated through the anterior system that is viewed as the executive system responsible for more voluntary and flexible cognitive functions such as attentional control (3). Based on the description of anterior system and its utility in research and clinical settings, Derry berry and Reed developed a scale named attentional control scale (ACS) to assess the differences in voluntary attentional control. The ACS comprises of 20 items that initially appeared to have 2 subscales: attentional focusing as the capacity to intentionally hold the attentional focus on desired channels, resisting unintentional shifting to irrelevant or distracting channels, and attentional shifting as the capacity to intentionally shift the attentional focus to desired channels, avoiding unintentional attention on particular channels (17). Factor analyses of the ACS in a study indicated 3 sub factors: (1) focus attention, (2) shift attention between tasks, and (3) flexibly control thoughts. 

The psychometric properties of the ACS have been assessed and reported in various studies in different countries. Factor structure analyses of the Dutch version of the ACS in a sample of 18 year- old children and adolescents have supported 2 factors, but omitted 2 items (Items 9 and 10) from the analysis, with internal consistency of α = 0.70 for focusing scale and α = 0.63 for shifting scale (18). A study on the Polish version of the ACS, using factor analysis, revealed 1-factor solution and then 3, 4, and 5 factor solutions using varimax and oblimin rotations. However, the 1-factor solution (KMO =.88, the total variance explained 35.4%) and 3-factor solution (KMO =.87, the total variance explained 47.8%) emerged as most suitable for psychological interpretation. Three factors were identified as follow: (1) attentional focusing, (2) attentional shifting, and (3) divided attention (19). Another study on the Icelandic version of the ACS on undergraduate students, using confirmatory factor analysis, yielded 2 factors of focusing and attention, which explained 35.13% of the variance. Furthermore, confirmatory factor analysis revealed a reasonable fit of this 2-factor model (20). The result of this study is in line with another research that showed 2- factor structure of the ACS using exploratory factor analysis, which explained 29.01% of the variance. Moreover, confirmatory factor analysis revealed a superior fit compared to the Icelandic version. Furthermore, internal consistency of the subscales were adequate, α = 0.82 for focusing and α = 0.71 for shifting (21). Attentional control was positively related to extraversion and negatively related to neuroticism. 

Considering the convergent and divergent validity of the ACS, the total score of the ACS was related to diary ratings of intrusive thoughts (22); moreover, shifting subscale was a significant predictor of depression, and focusing subscale was a significant predictor of anxiety (20). Also, attentional control was negatively related to behavioral inhibition system, negative affect, and maladaptive emotion regulation strategies, and it was positively related to behavioral approach system, positive affect, and adaptive emotion regulation strategies (19). Furthermore, attentional control was negatively correlated with worry and social anxiety disorder (21).

Attentional control is known as an emotion regulation strategy that is related to variety of anxiety and mood disorders (23), thus, understanding its relationship to psychopathology is helpful in improving o case conceptualization and treatment of various mental disorders, particularly refractory mental illnesses. Furthermore, it is the only questionnaire that assesses attentional control despite the suitable reliability and validity of the ACS in other countries, thus, these results could not be generalized to all the population of Iran, and its psychometric properties is unknown in the Iranian population. Therefore, the present study aimed at assessing the psychometric properties of the ACS (including factor structure, test retest reliability, and validity indexes) and determining the relationship between attentional control and anxiety (including generalized anxiety disorder and social anxiety disorder) and depression symptoms.

## Materials and Methods

*Participants*

This was a cross sectional study. Using quota sampling, 554 participants were recruited from a community sample in Tehran. Participants were classified according to statistical data obtained from the statistical center of Iran. Data were collected according to age, activity status, and education level, so our sample had the same proportions of individuals as the entire population with respect to aforementioned phenomena. Because the main aim of the present study was to assess the reliability and validity of the ACS, a large sample size was selected, (24). Data clean up and omitting 30 outliers using Mahalabonis and other methods to detect the outliers revealed 524 participants (46.4% females and 53.6% males). The mean age of the participants was 35.24 (SD =10.71) with 20 as the minimum and 60 as the maximum age. With respect to marital status, 31.7% of the participants were single, 65.5% married, and 2.9% were divorced. Of the participants, 14.9% did not have a high school diploma, 41.6%, 29.4%, 11.3%, and 2.9% held diploma, a bachelor’s degree, a master’s degree, and doctoral degree, respectively. Considering the activity status, 50.2% of the participants were employed, 7.1%, 25%, 11.5%, and 6.3% were unemployed, housekeepers, students, and had income without having a job, respectively. The statistical results of the study variables are presented in Table 1.

*Measures*

Attentional control scale was used as the main questionnaire. Furthermore, Five Facet Mindfulness Questionnaire, Emotion Regulation Questionnaire, Social Interaction Anxiety Scale, Beck Depression Questionnaire, Generalized Anxiety Disorder 7-Item Scale, Penn State Worry Questionnaire, and Rumination Response Questionnaire were used in the present study to evaluate convergent, divergent, and incremental validity of the ACS. 

Attentional Control Scale (ACS, (3): ACS is a self-report 20- item questionnaire rated on a 4 point Likert scale (1 = almost never to 4 = always) that assesses attentional control and attentional shifting. The internal consistency is reported to be as α = 0.88 (3) and test-retest reliability of the ACS items varies from 0.45 to 0.73 and it is 0.61 for the total score (19).

Five Facet Mindfulness Questionnaire (FFMQ, (25)): FFMQ is a self-report 39- item questionnaire rated on a 5 point Likert response scale ranging from 1 (never or very rarely true) to 5 (very often or always true). FFMQ includes 5 subscales: observing, describing, act aware, nonjudging, and nonreacting. Alpha coefficient of all facets of FFMQ reported to be as < 0.7 (26). The internal consistency of the subscales is 0.83, 0.91, 0.87, 0.87, and 0.75, respectively in the Chinese version of the questionnaire. Furthermore, the correlation between the subscales and BDI, STAI-State, STAI-Trait, and SCL-90 is agreeable (27). The internal consistency of the Five Facets and the total score of FFMQ in the Iranian population was α = 0.71, α = 0.83, α = 0.81, α = 0.73, α = 0.55, and α = 0.80; and the test-retest reliability was r = 0.84, r = 0.83, r = 0.68, r = 0.57, r = 0.71, and r = 0.80, respectively (28).

Emotion Regulation Questionnaire (ERQ, (29)): ERQ is a 10-item self-report questionnaire which is rated on a 7 point scale. ERQ consists of 2 subscales: reappraisal and suppression. The internal consistency of the 2 subscales has been reported 0.79 and 0.73, respectively (29). The psychometric properties of the Iranian version have good alpha coefficient for both subscales, α = 0.75 (30). Reappraisal subscale was used in the present study.

Social Interaction Anxiety Scale (SIAS, (31)): SIAS is a 20-item self-report questionnaire which is rated on a 5 point Likert scale ranging from 0 (not at all characteristic or true of me) to 4 (extremely characteristic or true of me). The internal consistency of SIAS in social phobia sample, community sample, and undergraduate sample has been reported to be as 0.86, 0.95, and 0.85, respectively (31). The psychometric properties among Iranian population has been demonstrated to have acceptable internal consistency of α = 0.90, and test-retest reliability of r = 0.79 (32).

Beck Depression Questionnaire (BDI-II (33)): BDI is a self-report 21- item questionnaire that assesses severity of depression disorder. Each item is scored from 0 to 3. The internal consistency of BDI is reported as 0.86 in psychiatric populations and 0.81 in nonpsychiatric populations. Test-retest reliability was also reported to be r = 0.86 (33). A study on 354 recovered depressed patients in Iran demonstrated internal consistency of α = 0/91 (34). 

Generalized Anxiety Disorder 7-Item Scale (GAD-7(35)): GAD-7 is a self-report 7- item questionnaire that rates the severity of generalized anxiety disorder and is rated on a 0 (not at all) to 3 (nearly every day) rating scale. The internal consistency and test-retest reliability were α = 0.92 and r = 0.83, respectively. The cutoff point of 10 has been identified with optimized sensitivity (89%) and specificity (82%) (35). Cronbach alpha of GAD-7 was α = 0.89 in the present research. 

 Penn State Worry Questionnaire (PSWQ, (36)): PSWQ is a 16-item self-report questionnaire that assesses worry on a 5 point response scale ranging from 1 (not at all typical of me) to 5 (very typical of me). It was demonstrated that the scale has very good internal consistency (α = 0.93) and high test-retest reliability (r = 0.74-0.93) (36). The psychometric properties of the Iranian version of the scale has been demonstrated to have high internal consistency (α= 0.88) and test-retest reliability (r = 0.79) (37).

The Ruminative Response Scale (RRS, (38): RRS is a 22- item self-report questionnaire that assesses the tendency to ruminate in response to depressed mood and is rated on a 4-point Likert-type scale that is varied from 0 to 3. Internal consistency (α = 0.89) and 5- month test- retest reliability have been reported acceptable (39). The internal consistency of the Iranian version was reported to be 0.81 (40).

*Procedure*

At first, the questionnaire was translated into Persian and back translated into English by bilingual experts in English language. Then, the two versions of the ACS were compared by another person who was adept in English language. At that point, the translated questionnaire was edited according to the comments, and the final translated ACS questionnaire was approved by 3 professors of University of Social Welfare and Rehabilitation Sciences who were familiar with attentional control concept. After preparing the final version of the Iranian ACS and obtaining approval from an institutional review board, we informed the participants about the goals of the study, ensured them about the confidentiality of their private information, and notified them they were not obliged to participate in the study. The participants filled in the questionnaire package, which included all the study questionnaires. 

*Data Analyses*

The primary goal of the present study was to conduct an exploratory factor analysis of a set of items assessing ACS. The second aim of the study was to assess test-retest reliability, internal consistency, convergent, divergent, and incremental validity of the ACS. It was hypothesized that rumination, worry, social anxiety, generalized anxiety, and depression were negatively and mindfulness and reappraisal positively correlated with attentional control. Furthermore, we hypothesized that attentional control could predict depression beyond its relationship with rumination and could predict social anxiety disorder beyond its relationship with anxiety and rumination. After checking the outliers and meeting the normality and linearity, factor analysis, bivariate correlation, and regression analysis were conducted to evaluate the hypotheses. SPSS Version 23 was used for data analysis. 

## Results

***Reliability Indexes***

Exploratory Factor Analyses: Exploratory factor analysis was performed. The chosen rotation method was oblimin rotation, as a previous study suggested that there was a correlation between the underlying factors (21). The Kaiser – Meyer – Olkin value was 0.82, which exceeds the recommended value of 0.7 (24). Bartlett’ s test of sphericity was significant and supported the factorability of the correlation matrix (41). Exploratory factor analysis resulted in 2 factors: focusing and shifting, with eigenvalues >1 explaining 20.30% and 10.63% of the variance, respectively (30.93% of the total variance). All factors loadings were higher than 0.3 except for Items 9, 15, and 20, which were not retained in EFA. Table 2 presents the items and respective factor loadings on the 2 primary underlying dimensions. Furthermore, focusing and shifting are almost moderately correlated with each other (Table 3). 

Internal Consistency and Test-Retest Reliability: Coefficient alpha for the focusing, shifting, and the total score of ACS were α = 0.78, α = 0.66, and α = 0.77, respectively that were almost in the range of acceptable recommended value for Cronbach’s alpha reliability except for the shifting subscale (42). To assess test- retest reliability, ACS was delivered to 57 participants in the present study after 14 days. Results of the test-retest reliability were as follow: focusing pre-post = 0.80, P<0.01, shifting pre-post= 0.076, P<0.01, and ACS.tot pre-post= 0.82, P<0.01. 

***Validity Indexes***

Convergent and Divergent Validity: The zero order correlations between the ACS and its subscales are demonstrated in Table 3. To provide an estimation of the effect size for statistically significant correlations, the r2 statistic are also provided in Table 4 as an indication of the percentage of common variance in the 2 measures. As hypothesized, reappraisal, as an emotion regulation strategy and mindfulness facets, had a positive relationship with focusing, shifting, and the total score of ACS. Worry, rumination, depression, generalized anxiety, and social anxiety symptoms all had negative relationships with focusing, rumination, and the total score of ACS. 

Incremental Validity: To examine the unique relationship between the subscales of ACS and symptoms of depression, generalized anxiety disorder, and social anxiety disorder, 3 hierarchical regression analyses were conducted. These analyses were done using the structure reported in previous studies that examined the ability of focusing and shifting subscales in evaluating the significant amount of variability in depression and anxiety symptoms above and beyond the established construct of rumination and worry (43). The first model examined the predictors of depression symptoms (Table 5). Rumination examined by RRS was entered as a predictor in the first step and depression symptoms examined by BDI were entered in the second step. Focusing, but not shifting, uniquely predicted depression symptoms. The second model examined the predictors of generalized anxiety symptoms (Table 5). Worry examined by PSWQ was entered as a predictor in the first step and generalized anxiety symptoms examined by GAD was entered in the second step. Focusing but not shifting uniquely predicted generalized anxiety symptoms. The third model examined the predictors of social anxiety symptoms (Table 5). Rumination and worry were entered as predictors in the first step, and social anxiety symptoms examined by SIAS was entered in the second step. Both focusing and shifting uniquely predicted social anxiety symptoms.

**Table 1:** The Descriptive Statistics of the Study Measured Variables

	**Minimum**	**Maximum**	**Mean**	**S.D**	**Skewness**	**Kurtosis**
ACS_(short)_	23	63	44.37	6.73	0.01	0.13
BDI	0	58	6.86	11	1.053	0.82
GAD	0	24	8.1	5.1	0.574	0.069
FFMQ	85	167	124.69	14.79	0.414	-0.047
Reappraisal	9	42	25.39	6.13	-0.181	-0.053
PSWQ	17	80	46.45	10.73	0.335	0.198
RRS	22	82	47.45	11.8	0.387	-0.002
SIAS	0	69	22.18	13.93	0.722	0.125

**Table2:** Exploratory Factor Analysis of the attentional control scale in Iranian Sample

**Items**	**Focusing**	**Shifting**	**r** ^a^
1. It is very hard for me to concentrate on a difficult task when there are noises around.	0.58	0.02	0.54
2. When I need to concentrate and solve a problem, I have trouble focusing my attention.	0.67	0.14	0.65
3. When I am working hard on something, I still get distracted by events around me.	0.61	0.15	0.60
4. My concentration is good even if there is music in the room around me.	0.25	0.38	0.44
5. When concentrating, I can focus my attention so that I become unaware of what's going on in the room around me.	0.13	0.37	0.39
6. When I am reading or studying, I am easily distracted if there are people talking in the same room.	0.57	0.17	0.59
7. When trying to focus my attention on something, I have difficulty blocking out distracting thoughts.	0.63	0.13	0.61
8. I have a hard time concentrating when I am excited about something.	0.52	0.11	0.54
9. When concentrating I ignore feelings of hunger or thirst.	-0.2	0.23	-0.02
10. I can quickly switch from one task to another.	-0.09	0.31	0.33
11. It takes me a while to get really involved in a new task.	0.35	0.16	0.41
12. It is difficult for me to coordinate my attention between the listening and writing required when taking notes during lectures.	0.52	0.27	0.55
13. I can become interested in a new topic very quickly when I need to.	0.02	0.47	0.47
14. It is easy for me to read or write while I'm also talking on the phone.	0.18	0.41	0.45
15. I have trouble carrying on two conversations at once.	0.17	0.14	0.2
16. I have a hard time coming up with new ideas quickly.	0.36	0.16	0.4
17. After being interrupted or distracted, I can easily shift my attention back to what I was doing before.	0.2	0.52	0.51
18. When a distracting thought comes to mind, it is easy for me to shift my attention away from it.	0.24	0.41	0.44
19. It is easy for me to alternate between two different tasks.	0.22	0.63	0.59
20. It is hard for me to break from one way of thinking about something and look at it from another point of view.	0.19	-0.05	0.09

**Table3:** Descriptive Statistics and Scale and Factor Intercorrelations in attentional control scale

	**M**	**SD**	**Skewness**	**Kurtosis**	**1**	**2**
1.Focusing	25.28	4.59	-0.38	-0.16	(0.78)	0.30**
2.Shifting	19.08	3.88	0.35	0.20	0.18	(0.66)

Note: Alpha reliabilities of the subscales are found on the diagonal. Correlation coefficients for scale values appear below the diagonal, and factor correlations appear above the diagonal.

** P<0.01.

**Table4:** Correlations of the Subscales of attentional control scale and Its Total Score and Criterion Measures

		**r**			**R** ^2^	
Variable	ACS (short)	Focusing	Shifting	ACS (short)	Focusing	Shifting
BDI	-0.29**	-0.35**	-0.1*	0.08	0.12	0.01
GAD	-0.3**	-0.37**	-0.1*	0.09	0.13	0.01
FFMQ	0.52**	0.44**	0.4**	0.28	0.19	0.16
Reappraisal	0.22**	0.11**	0.25**	0.04	0.01	0.06
PSWQ	-0.43**	-0.49**	-0.19**	0.19	0.24	0.03
RRS	-0.3**	-0.37**	-0.09*	0.06	0.14	0.01
SIAS	-0.48**	-0.46**	-0.31**	0.23	0.21	0.09

ACS (short): ACS full scale, with reduced items (17 items).

** P<0.01

* P<0.05

**Table 5:** Summary of Hierarchical Regression Analysis Examining Focusing and Shifting as Predictors of Depressive Symptoms (BDI), Generalized Anxiety Symptoms (GAD), and Social Anxiety Symptoms (SIAS)

	**B**	**SE B**	**β**	**t**
**Dependent variable: depression**				
**Step 1** **R** ^2^ **=0.32**				
**Constant**	-11.38	1.64		
**RRS**	0.53	0.03	0.56	15.81^**^
**Step 2** **R** ^2^ **=0.34**				
**Constant**	1.51	3.6		
**RRS**	0.47	0.03	0.5	13.26^**^
**Focusing**	-0.38	0.09	-0.16	-4.02^**^
**Shifting**	0.02	0.1	-0.008	-0.21
**Dependent variable: Generalized Anxiety disorder**				
**Step 1** **R** ^2^ **=0.31**				
**constant**	-4.46	0.83		
**PSWQ**	0.27	0.01	0.56	15.53^**^
**Step 2** **R** ^2^ **=0.32**				
**Constant**	-0.13	1.96		
**PSWQ**	0.24	0.02	0.5	12.16^**^
**focusing**	-0.15	0.04	-0.13	-3.1^**^
**Shifting**	0.04	0.05	0.03	0.94
**Dependent variable: social Anxiety disorder**				
**Step 1** **R** ^2^ **=0.29**				
**Constant**	-14.01	2.48		
**PSWQ**	0.47	0.05	0.36	8.35^**^
**RRS**	0.29	0.05	0.25	5.47^**^
**Step 2** **R** ^2^ **=0.37**				
**constant**	22.18	5.31		
**PSWQ**	0.32	0.05	0.25	5.61^**^
**RRS**	0.32	0.05	0.22	5.29^**^
**Focusing**	-0.6	0.12	-0.19	-4.75^**^
**Shifting**	-0.6	0.13	-0.18	-4.95^**^

## Discussion

In line with previous studies indicating 2 separate subscales (3, 20, 21), the exploratory factor analysis in the present study yielded 2 factors: the focusing factor, assessing the ability to keep attention when facing with distractors or external stimuli irrelevant to the major task at hand (44), and the shifting factor, assessing the ability to shift between 2 tasks. Focusing items were more homogenous, explaining 20.30% of the total 

variance, with acceptable internal consistency (α = 0.78); and in contrast, the shifting subscale items were 

more heterogeneous, explaining 10.63% of the total variance, with lower and not agreeable internal consistency (α = 0.66). Items 9, 15, and 20 were 

deleted from the remaining analysis because of loading lower than 0.3. Results of the loading items on the subscales were different from some studies (3, 20). However, there was a substantial overlap between the factor structures in the present study and that of Judah et al., Almost all items were loaded on the same factors except Items 4, 5, 11, 14, and 16 that were not retained in the EFA in study of Judah et al. However, in the present study, aforementioned items were included in the remaining analysis. Confirmatory factor analysis conducted to evaluate the fitness indices of the empirical model in a study by Judah et al. suggested good fit to the data, χ2(53) = 78.96, p = .01, CFI = .96, TLI = .95, RMSEA = 0.05, AIC =152.96 (21). Focusing and shifting factors were moderately correlated (r = 0.52, P<0.01), which is in line with previous research (20, 21). Furthermore, there are some discords among the studies examining the factor structure of the ACS, and as the present study’s results demonstrated, more explorations are needed to inspect different structures of the ACS, and to add, or delete some items, or develop new tools for assessing the broad aspects of attentional control. To use the ACS in the Iranian population, we recommend computing items related to each factor indicated in EFA (Table 2); and as the shifting factor does not possess enough reliability, we recommend using the full scale score along with its subscales for various analyses.

The second aim of the present study was to evaluate the validity of attentional control using measures that theoretically or empirically are associated with the construct of attentional control. Anxiety and depression vulnerabilities are characterized by attentional bias to threat. Attentional bias modification trainings have reduced the attentional bias to threat (14). In line with these findings, depression, generalized anxiety disorder, social anxiety disorder, worry, and rumination were used to evaluate the divergent validity of attentional control. Results indicated acceptable negative correlation between the variables. Consistent with the findings of the present research, fear of performance negatively affects individuals with low attentional control. Moreover, attentional control as a self-regulation strategy acts as a buffer in performance anxiety (45). Moreover, impaired attentional control and high attentional bias towards threat in social anxiety disorder have been highlighted in several studies (46, 47). Besides, the relationship between anxiety and depression symptoms and attentional control have been indicated through different methodologies using a wide range of questionnaires or tools (16, 48). Also, the inability to control worry related negative thoughts and rumination is related to deficiency in attentional control (12, 13). To evaluate the convergent validity of attentional control, mindfulness facets and reappraisal were used. These constructs were moderately and positively associated with attentional control, which is in line with the findings demonstrating the moderating role of attentional control in the relationship between difficulties in emotion regulation and distress tolerance (2), correlation between attentional control, emotion regulation strategies (19), and mindfulness (49).

The final goal of the present study was to evaluate the incremental validity of attentional control full scale and focusing and shifting subscales in predicting depression, generalized anxiety disorder, and social anxiety disorder. Attentional control full scale and focusing, not shifting, predicted depression and generalized anxiety symptoms after controlling for the shared variance between anxiety and depressive symptoms, and worry and rumination. Attentional control full scale, shifting, and focusing predicted social anxiety symptoms beyond the shared relationship between anxiety and worry and rumination. The findings are inconsistent with previous findings showing the unique relationship of anxiety with focusing and unique relationship of depression with shifting (20, 21). This discrepancy may be due to the low reliability of the shifting subscale in the present study. Furthermore, there was a moderate to high correlation between the shifting and focusing subscale in the present study, and some studies have suggested to treat ACS as a solitary dimension of effortful emotional control (19), and this may indicate that using attentional control full scale may be more useful and reliable in different analyses. 

## Limitations

Concerning limitations of the present study, a community sample was used in the present study, so it was unclear to what extent the present findings could be generalized to broader and clinical population. Furthermore, all study variables including attentional control were assessed by self-report questionnaires, and as these types of scales may be biases, future research should use more precise procedures or tools, performance based tasks (43), and physiological measures to evaluate the attentional control. To our knowledge, this was the first study to evaluate the psychometric properties of ACS in Iranian population, so we recommend future studies to repeat this EFA in another sample and conduct CFA to assess the fitness of the multicomponent nature of attentional control. Finally, attentional control is a construct whose factors are not clearly known, so other studies are required to add other items to increase the reliability and validity of its factors. Furthermore, because attentional control and its subscales, especially focusing, were related to anxiety and depression, some investigations that illuminated the various pathways of attentional control and its different roles in psychopathology may turn on dark points in psychopathologies and treatments.

## Conclusion

Attentional control scale is a sole self- reported measure that assesses attentional control and it has suitable psychometric properties to be used in Iranian population. Furthermore, attentional control can be regarded as an emotion regulation strategy or as a mechanism that may contribute to anxiety and depression and may be a protective factor, and can be enhanced in psychotherapies instead of dealing with repetitive negative thoughts (like worry and rumination) directly.
